# Marine Bacteria, A Source for Alginolytic Enzyme to Disrupt *Pseudomonas aeruginosa* Biofilms

**DOI:** 10.3390/md17050307

**Published:** 2019-05-24

**Authors:** Said M. Daboor, Renee Raudonis, Alejandro Cohen, John R. Rohde, Zhenyu Cheng

**Affiliations:** 1 Department of Microbiology and Immunology, Dalhousie University, Halifax, Nova Scotia, NS B3H 4R2, Canada; r.raudonis@dal.ca (R.R.); john.rohde@dal.ca (J.R.R.); 2 Department of Biochemistry and Molecular Biology, Dalhousie University, Halifax, Nova Scotia, NS B3H 4R2, Canada; alejandro.cohen@dal.ca; 3 National Institute of Oceanography and Fisheries, Cairo 11516, Egypt

**Keywords:** marine bacteria, *Pseudomonas aeruginosa*, biofilm, alginate lyase

## Abstract

*Pseudomonas aeruginosa* biofilms are typically associated with the chronic lung infection of cystic fibrosis (CF) patients and represent a major challenge for treatment. This opportunistic bacterial pathogen secretes alginate, a polysaccharide that is one of the main components of its biofilm. Targeting this major biofilm component has emerged as a tempting therapeutic strategy for tackling biofilm-associated bacterial infections. The enormous potential in genetic diversity of the marine microbial community make it a valuable resource for mining activities responsible for a broad range of metabolic processes, including the alginolytic activity responsible for degrading alginate. A collection of 36 bacterial isolates were purified from marine water based on their alginolytic activity. These isolates were identified based on their 16S rRNA gene sequences. *Pseudoalteromonas* sp. 1400 showed the highest alginolytic activity and was further confirmed to produce the enzyme alginate lyase. The purified alginate lyase (AlyP1400) produced by *Pseudoalteromonas* sp. 1400 showed a band of 23 KDa on a protein electrophoresis gel and exhibited a bifunctional lyase activity for both poly-mannuronic acid and poly-glucuronic acid degradation. A tryptic digestion of this gel band analyzed by liquid chromatography-tandem mass spectrometry confirmed high similarity to the alginate lyases in polysaccharide lyase family 18. The purified alginate lyase showed a maximum relative activity at 30 °C at a slightly acidic condition. It decreased the sodium alginate viscosity by over 90% and reduced the *P. aeruginosa* (strain PA14) biofilms by 69% after 24 h of incubation. The combined activity of AlyP1400 with carbenicillin or ciprofloxacin reduced the *P. aeruginosa* biofilm thickness, biovolume and surface area in a flow cell system. The present data revealed that AlyP1400 combined with conventional antibiotics helped to disrupt the biofilms produced by *P. aeruginosa* and can be used as a promising combinational therapeutic strategy.

## 1. Introduction

Bacterial biofilms represent the dominant bacterial growth lifestyle in different habitats, including natural and clinical environments [1,2,3,4,5]. Bacterial cells switch between the free-living planktonic mode and biofilm growth mode through multiple stages that include adhesion to a substratum, increasing their aggregation into micro-colonies and maturation of biofilms via matrix formation through secretion of extracellular polymeric substances [6,7,8]. It is estimated that 65%–80% of human infections involve biofilms, causing a huge burden on healthcare systems globally [5,9,10].

*Pseudomonas aeruginosa* is an opportunistic pathogen with the ability to colonize a wide range of hosts and/or substrates and produce biofilms. *Pseudomonas aeruginosa* biofilms are commonly found in patients with cystic fibrosis (CF) and chronic obstructive pulmonary disease with persistent infections. This bacterium also forms biofilms on medical devices, such as central venous and urinary catheters, stents, orthopedic prosthesis, and mechanical heart valves, causing infections that are extremely difficult to treat, and thus have a massive impact on healthcare funding [11,12,13,14].

*Pseudomonas aeruginosa* cells within biofilms demonstrate a slow growth rate that endures many therapeutic antimicrobial agents due to substantially diminished antibiotic diffusions [15,16], and causes weakened cell-mediated host defenses [17,18,19]. As a result, the clinical management of those biofilm-ensconced bacteria has become intractable [11,20]. The failure to eradicate the pathogen from the lung often leads to increased possibility of chronic infections that are connected by acute exacerbations of disease and inflammation, resulting in lung failure and high morbidity among patients [21,22,23,24,25,26]. However, *P. aeruginosa* biofilm infections go beyond CF diseases, especially with diabetic patients. These patients are prone to developing chronic wounds that lack sufficient healing capabilities, thus resulting in biofilm-associated, long-term infections, which cause financial burdens for the patients [9,27,28].

The treatment of *P. aeruginosa* established biofilms with enzymes that degrade the biofilm matrix have been widely used as an alternative treatment approach for bacterial infection. The levanase SacC [29], DNase (NucB) that hydrolyzes the eDNA within the extracellular matrix [30], and glycoside hydrolase that hydrolyzes the biofilm exopolysaccharide [31] have all been shown to act against *P. aeruginosa* biofilm. The overproduction of the polysaccharide alginate is the major cause for the mucoid phenotype of *P. aeruginosa* that is associated with a decline in lung function. Cultures of CF patients’ sputum showed high amounts of alginate and a massive production of antibodies against alginates including IgG and IgA classes [4,11]. Moreover, alginate is a major component of *P. aeruginosa* biofilms, making it an attractive target to limit biofilm-associated infections of this extremely important bacterial pathogen. Disrupting alginate structure will potentially promote the killing rate of bacterial pathogens by human immune cells and improve antipseudomonal antibiotic efficacy. The use of alginate lyase was considered as an approach to improve treatment for bacterial infections within biofilm by breaking down the main part of biofilm matrix [32]. Marine bacteria are known to be a potential source for alginate lyase, due to the high possibility of encountering alginate that is rich in their environment, as it is a cell wall component of brown algae [33]. *Pseudoalteromonas*, a marine bacterium that produces exoproducts including alginate lyase, demonstrated antibacterial activity against many pathogenic bacteria, such as *Salmonella enterica*, *Staphylococcus aureus*, *P. aeruginosa*, *Escherichia coli* and *Enterococcus faecalis* [34] and antibiofilm activity against *S. enterica*, *P. aeruginosa* and *E. coli* [35]. In this work, we purified alginate lyase from a *Pseudoalteromonas* isolate. Importantly, we showed that the purified alginate lyase was able to disrupt the established biofilm of *P. aeruginosa*. This study will facilitate the development of a combinational therapeutic strategy, by using alginate lyase and antibiotics, to tackle the problem of *P. aeruginosa* biofilm-associated infections.

## 2. Results

### 2.1. Isolation of Bacterial Strains with Alginolytic Activity

**Figure 1:**
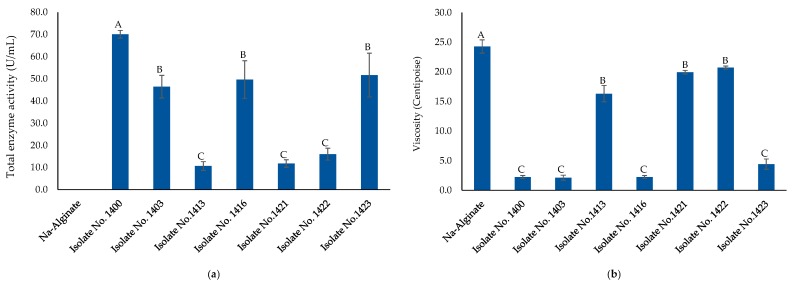
Alginate lyase activity produced by different bacterial strains. (**a**) Total enzyme activity represented as unit per mL, where each one-unit enzyme is defined as the amount of enzyme required to increase the absorbance at 235 nm by 0.01 per minute. (**b**) Viscosity reduction of selection medium with 1.8% sodium alginate after 24 h incubation measured in centipoise, a unit of absolute viscosity. Error bars represent standard deviation of three independent experiments. The blank is designated as Na-alginate (sodium alginate). Different letters indicate a statistically significant difference between groups (*p* < 0.05).

**Figure 2:**
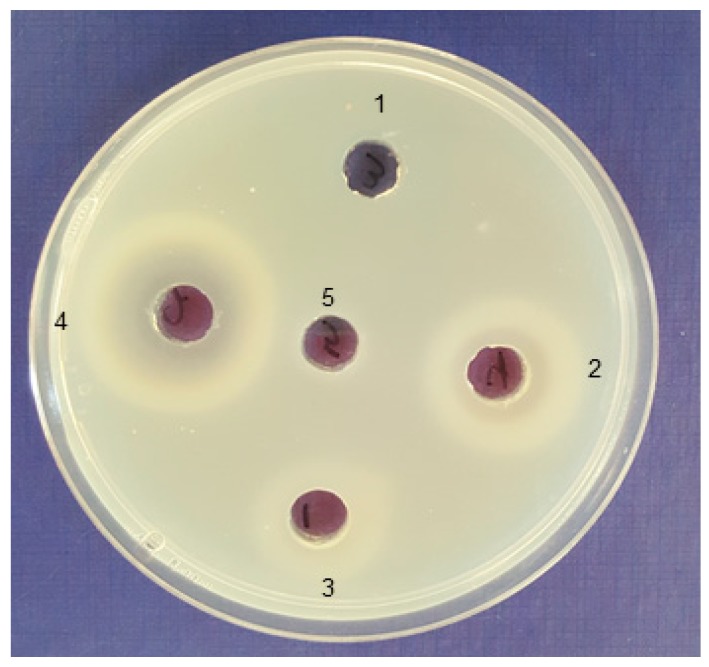
Bifunctional activity of alginate lyase produced by isolated marine bacteria. Enzymes acting against poly-mannuronate (poly-M) show a white halo due to gelation of the degradation products caused by the reaction of lyase with poly-M and enzymes acting against poly-guluronate (poly-G) show a white ring. The lyase activity of the following isolates’ supernatants are shown: 1, *Cellulophaga* sp. 1423; 2, *Pseudoalteromonas* sp. 1422; 3, *Pseudoalteromonas* sp. 1416; 4, *Pseudoalteromonas* sp. 1400; and 5, well is filled with 1.8% alginate solution in 20 mM Tris-HCl buffer as a blank.

A total of 36 bacterial strains with alginate lyase activity were isolated after enrichment on bacterial agar plates containing sodium alginate and selected for 16S rRNA sequencing (GenBank accession numbers MK377259 through MK377294). The 16S rRNA sequence analysis revealed that most obtained strains belong to five genera, *Celeribacter*, *Cellulophaga*, *Pseudoalteromonas*, *Shewanella*, and *Vibro*. As shown in Table 1, *Pseudoalteromonas* spp. represented 52% of the identified isolates (19 out of 36 isolates). Alginate lyase activity was determined based on the hydrolytic clearance zone diameter produced after adding the Lugol’s solution to alginate plates. Seven bacterial isolates (No. 1400, 1403, 1413, 1416, 1421, 1422 and 1423) that produced the highest alginate lyase activity by exhibiting the largest clear zones are highlighted in bold in Table 1. Isolate 1400 produced the largest clearing hydrolytic zone with a diameter of 30.0 ± 1.4 mm. The 16S rRNA gene sequence of the isolate 1400 showed highest identity (99%) to a *Pseudoalteromonas tetraodonis* strain GFC. Based on these results, isolate 1400 was designated as *Pseudoalteromonas* sp. 1400. The cell-free supernatant of medium that had been used to culture this strain contained a total alginolytic activity of 70.09 ± 1.72 U/mL (Figure 1a) and significantly (*P* < 0.0001) reduced sodium alginate solution viscosity to 2.23 ± 0.25 centipoise with 90.81% liquefaction after 24 h incubation (Figure 1a), while sodium alginate solution (the blank) viscosity was 24.28 ± 1.12 centipoise (Figure 1b). The plate assay technique was used to detect the enzyme substrate specificity. After covering the alginate agar plate with 1 M CaCl_2_ solution, enzymes acting on poly-mannuronate (poly-M) will show a white halo due to gelation of the degradation products caused by the reaction of lyase with poly-M. Meanwhile, poly-guluronate (poly-G) substrate will show a white ring, due to the depolymerization of alginate to uronic acid. Appearance of both phenomena, as clearly observed by *Pseudoalteromonas* sp. 1400 supernatant indicated that its alginolytic enzyme possessed substrate specificity for both poly-G and poly-M (Figure 2).

### 2.2. Antibiofilm Activity of Cell-Free Supernatants of Seven Isolates

**Figure 3:**
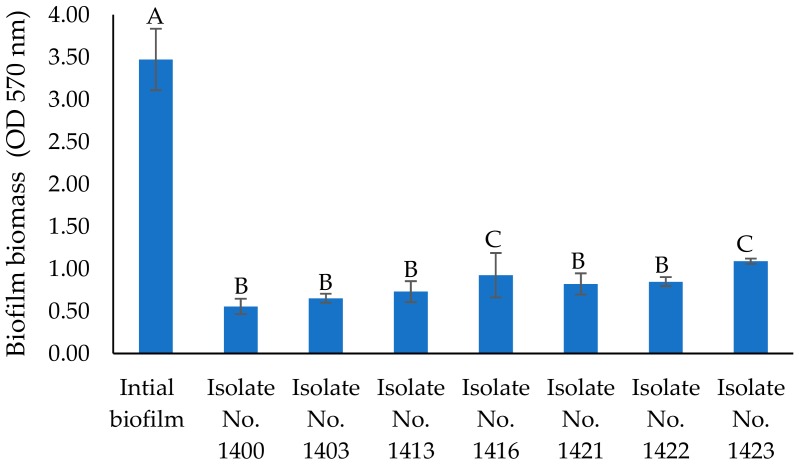
Biofilm biomass formed after 48 h in 96-well microplates, treated with different cell-free supernatants for 24 h at 37 °C under static conditions. Error bars represent standard deviation (SD) of three independent experiments. Different letters indicate statistically significant differences between groups (*p* < 0.05).

The cell-free supernatants of seven bacterial strains (listed in bold in Table 1 above) were screened for antibiofilm activity against *P. aeruginosa* strain PA14. Pre-formed biofilms (48 h) in a 96-well microplate were treated with the cell-free supernatant of each bacterial strain or medium control. The remaining biofilm was stained using crystal violet and the relative biomass was measured using absorbance at 570 nm. Our data revealed that the supernatants from all isolates disrupted the established PA14 biofilm (Figure 3). *Pseudoalteromonas* sp. 1400 supernatant treatment led to a 69% reduction in PA14 biofilm biomass. There were no significant changes (*P* ≥ 0.05) for the majority of the tested supernatants in biofilm disruption efficiencies between the 24 h and 48 h (Figure S1) treatment period.

### 2.3. Alginate Lyase Production and Purification

**Figure 4:**
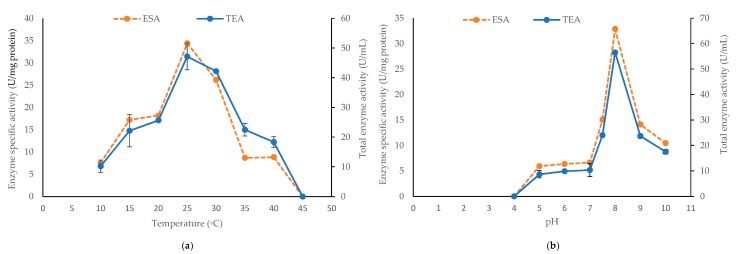
The alginate lyase enzyme specific activity (ESA) and total enzyme activity (TEA) produced by *Pseudoalteromonas* sp. 1400 after 24 h incubation at 150 rpm with (**a**) different temperatures and (**b**) different pH values. One-unit enzyme is defined as the amount of enzyme required to increase the absorbance at 235 nm by 0.01 per minute. The error bars for TEA represented the SD values of three independent experiments as means ± SD (*n* = 3).

Isolate 1400 was selected for subsequent enzyme purification and characterization because it demonstrated the best and highest alginate lyase activity as shown by having the largest clearance zone (Table 1) and enzyme production measured in U/mL (Figure 1a). First, we set to optimize the growth conditions (including temperature and medium pH), under which *Pseudoalteromonas* sp. 1400 produced the highest alginate lyase activity in the supernatant. We recorded a specific enzyme activity of 34.4 U/mg protein with 47.17 ± 4.45 U/mL total activity when incubated at 25 °C. This activity was significantly reduced (*P* < 0.001) when incubated at lower temperature, i.e., 10 °C. At 35 °C and 40 °C, only 8.7 U/mg protein was released. Meanwhile, no alginate lyase activity was detected with growth performed at 45 °C (Figure 4a). On the other hand, *Pseudoalteromonas* sp. 1400 grown at pH 8.0 released the highest amount of alginate lyase with specific activity of 32.85 U/mg protein and 56.5 ± 0.71 U/mL total activity, whereas we noticed that the lowest specific enzyme activity (5.92 U/mg protein) was recorded at pH 5.0 and pH 6.0. As shown in Figure 4b, there was no lyase activity detectable at pH 4.0.

Based on the optimal alginate lyase production conditions, we set to purify this enzyme (AlyP1400) from *Pseudoalteromonas* sp. 1400 grown in 9 L of selection medium with 1.8% sodium alginate for 24 h at pH 8 and a temperature of 25 °C. Table 2 shows the detailed information of the implemented multi-step purification procedures. The crude AlyP1400 exhibited a specific activity of 17.67 ± 0.018 U/mg protein. This value increased to 106.47 ± 1.4 U/mg protein after fractionation II with ammonium sulfate (80%), while the purified AlyP1400 specific activity that resulted from Bio-Gel column chromatography (Sephadex G-100) was 342.16 ± 3.84 U/mg protein.

**Figure 5:**
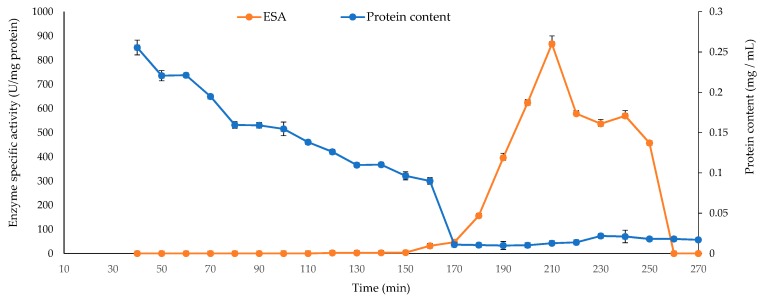
Protein content and specific enzyme activities for AlyP1400 obtained from the Sephadex G100. One-unit enzyme defined as the amount of enzyme required to increase the absorbance at 235 nm by 0.01 per minute. Error bars represented the SD values of three independent experiments as means ± SD (*n* = 3).

The achieved enzyme purification fold was 19.36 after bio-gel column chromatography, with a yield of 0.5%. During the first 120 minutes of purification time, no lyase activity was observed with the collected fractions through the bio-gel column chromatography eluate with 0.2 M sodium chloride. The fractions recovered after 180 minutes, and the eluate from 0.3 to 0.4 M sodium chloride showed a specific activity value between 31.8 ± 2.6 and 867 ± 32.75 U/mg protein. Increasing the purification time beyond 220 minutes significantly reduced the alginate lyase activity (Figure 5). After buffer exchange, we stored the purified AlyP1400 in 20 mM Tris-HCl (pH 7.5) at 0.7 mg protein/mL final concentration at −20 °C until further use.

### 2.4. Biochemical Properties of Purified AlyP1400

**Figure 6:**
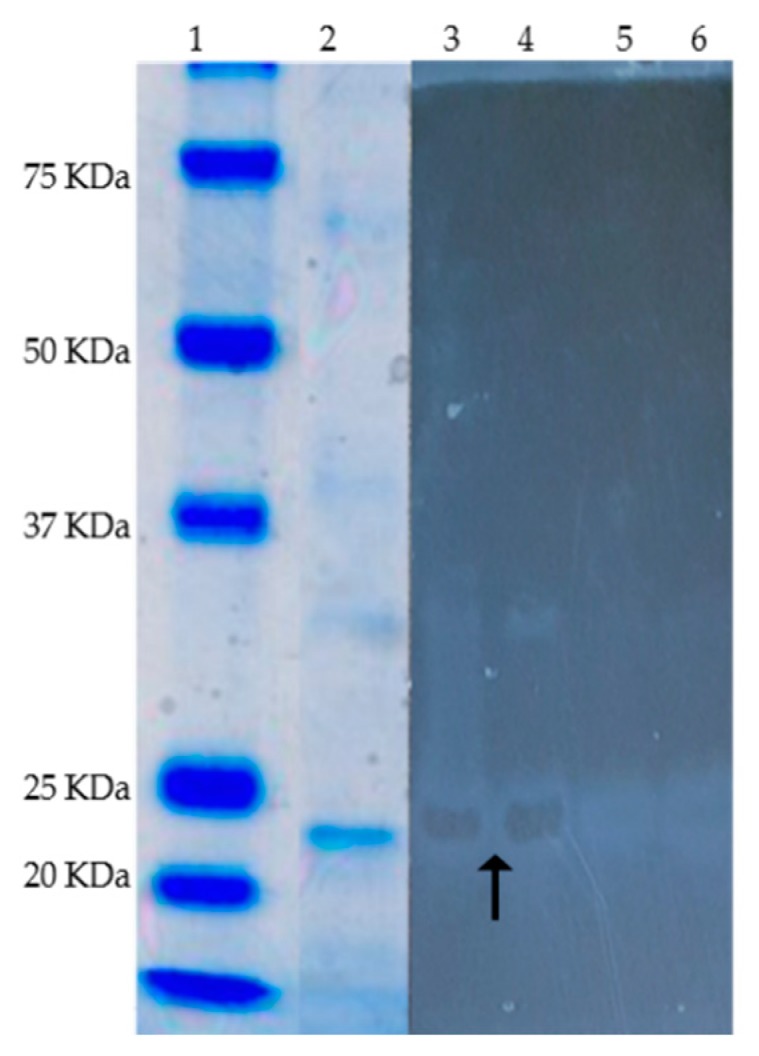
SDS-PAGE (Coomassie blue staining) and zymogram (active staining for alginate lyase) of the purified alginate lyase (AlyP1400) from *Pseudoalteromonas* sp. 1400. Lanes are as follows, 1, protein markers; 2, 3 and 4 purified alginate lyase (black arrow show the alginate hydrolysis in 3 and 4), 5 and 6 negative control, 0.1% alginate solution in 20 mM Tis-HCl buffer.

**Figure 7:**
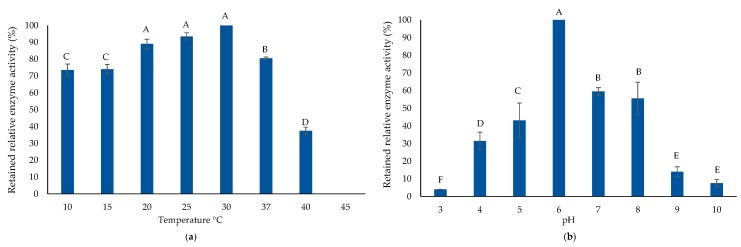
AlyP1400 biochemical characterization. (**a**) Thermal enzyme stability and (**b**) the pH stability of the purified AlyP1400 are illustrated. Error bars represent SD of three independent experiments. Different letters indicate statistically significant differences between groups (*p* < 0.05).

AlyP1400 was active for both poly-M and poly-G degradation, indicating that it is a bifunctional alginate lyase (data not shown). SDS-PAGE and zymogram analysis illustrated that the enzyme had a molecular mass of ~23 KDa (Figure 6). To characterize both the thermal and pH stability of the purified AlyP1400, the enzymatic activity was measured after incubation at different temperatures and then with buffers that had a wide range of pH values. AlyP1400 activity revealed stability at low temperature (10 °C). The maximum activity was recorded at 30 °C, which dramatically decreased when temperature increased to 40 °C. The enzyme was completely inactivated when incubated at 45 °C (Figure 7a). The highest AlyP1400 activity was recorded when the enzyme was incubated in a buffer with pH 6.0 and about 60% of its activity was retained at pH 7.0. This activity decreased significantly (*P* < 0.0001) when pH changed towards either alkaline or acidic conditions. AlyP1400 lost about 43% of its activity when incubated in buffer with pH 5.0, while more than 90% of the activity was lost when the AlyP1400 was incubated in pH 3.0 or pH 10.0 buffers (Figure 7b).

### 2.5. Liquid Chromatography Tandem-Mass Spectrometry (LC-MS/MS) Enzyme Analysis

The AlyP1400 band identified in the SDS-PAGE with alginate lyase activity was excised, subjected to trypsin digestion and analyzed by LC-MS/MS. A protein identification search was performed using Proteome Discover 2.2 software against the broad National Centre for Biotechnology Information (NCBI) Pseudoalteromonadaceae (Taxonomy ID 267888) database in consideration of all possible variants within this bacterial family. The cRAP (Common Repository of Adventitious Proteins) database was also selected to account for common background contaminant proteins typically found in proteomics experiments. A protein named alginase (Entry ACB87607, *Pseudoalteromonas* sp. SM0524), containing a carbohydrate binding domain and an alginate lyase region, was identified as the top scoring protein found in this gel band, excluding a few human keratin protein hits. Seven alginate lyase peptides, including four unique peptides were identified from the LC-MS/MS data (Figure S2). These peptide sequences were further searched using BLAST at the NCBI database. The results showed that the peptide sequencing matched some alginate lyases in the database. The identified N-terminal sequences of AlyP1400 are related to the following: the alginate lyases from *Alteromonas* sp. 272; the extracellular alginate lyase produced by *Pseudoalteromonas* sp. IAM14594; a β-D-mannuoronate lyase from several strains of *P. agarivorans*, *Pseudoalteromonas* sp. NW 4327, *Pseudoalteromonas* sp. P1-25, and *P. telluritirucens*; and a polysaccharide lyase family 7 protein produced from *Gilvimarinus polysaccharolyticus*. These finding suggest that AlyP1400 has a higher identity to the alginate lyases in polysaccharide lyase family 18 Aly-sj02 R.

### 2.6. Inhibitory Effect of AlyP1400 against P. aeruginosa PA14 Biofilm

**Figure 8:**
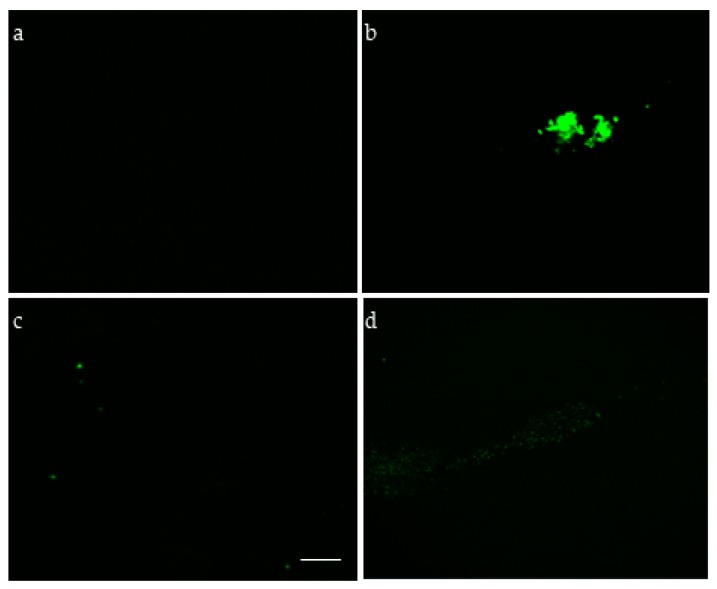
Alginate production by *P. aeruginosa* PA14 grown under biofilm conditions. Fluorescent microscopic images of bacterial cells incubated with MAb F429 and a secondary antibody conjugated with Alexa Fluor 488 are shown. (**a**) Non-alginate producer *E. coli* TOP10, (**b**) *P. aeruginosa* PA14, (**c**) *P. aeruginosa* PA14 control without the primary MAb F429, (**d**) PA14 with AlyP1400 enzyme. Scale bar, 10 µm.

We first used an immunofluorescence experiment to determine alginate production by *P. aeruginosa* PA14 within biofilms after 48 h that used specific antibodies (Figure 8). PA14 produced alginate on minimal medium, M63, and showed a clear reaction with the *P. aeruginosa* alginate-specific monoclonal antibody (MAb F429) (Figure 8b). The biofilms grown in M63 in the presence of AlyP1400 (60 µg/mL) was shown to reduce antibody binding to alginate, with little to no alginate staining, indicating the hydrolytic activity of AlyP1400 against the alginate produced by PA14 (Figure 8d). Controls lacking primary antibody (Figure 8a) and non-alginate producer *E. coli* (cloning strain TOP10) (Figure 8c) were negative for immunofluorescence signals.

To further characterize the activity of the purified AlyP1400 against *P. aeruginosa* PA14 biofilms, we used a flow-cell biofilm cultivation system to examine the effect of AlyP1400 alone, and in combination with carbenicillin (CB) and ciprofloxacin (Cip) on PA14 biofilms architecture under a continuous flow condition. The 48-h old biofilms pre-formed in a flow-cell chamber were treated for 2 h and then stained with viability dyes: Syto 61 Red that stains all bacterial cells and emits red fluorescence; while Sytox Green stains cells with permeabilized membranes, damaged or dead cells with compromised membranes, and emits green fluorescence. The non-treated control sample revealed a stable biofilm with a characteristic architecture of mature biofilm and very few to no patches of cells that stained green, indicating that the vast majority of biofilm was living bacteria (Figure S3a). The control biofilm surface had an average thickness of 37.2 ± 3.74 µm, biovolume of 10.27 ± 3.77 µm^3^/µm^2^, and surface area of 5.0 ± 3.5 µm^2^ (Table 3). After CB (100 µg/mL) or Cip (12 µg/mL) treatment (without alginate lyase), we found that the antibiotic-treated biofilms were disrupted to non-uniform structures (Figure S3b,c). This was consistent with the significantly reduced average biofilm thickness, which was 27.9 ± 4.45 µm and 27.7 ± 4.14 µm for CB- and Cip-treated samples, respectively (Table 3). The aggregates of membrane compromised cells (green color) were observed in the peripheral area of the antibiotics-treated biofilms, remaining sessile to the glass surface with average biovolumes and surface areas comparable to non-treated control (Table 3). This suggested that the antibiotics only affected the top layer of the biofilms and were not efficient in significantly reducing viable cells protected within the inner layers of the biofilms. Standalone treatment with AlyP1400 (60 µg protein/mL) significantly reduced the biofilm thickness to 25.3 ± 2.41 µm, biovolume to 7.71 ± 3.79 µm^3^/µm^2^, and surface area to 2.6 ± 1.6 ×10^5^ µm^2^ (Table 3).

Next, the combinational effects for AlyP1400 and conventional antibiotics was tested. The combination of AlyP1400 with either CB or Cip significantly increased the biofilm inhibitory activity of each antibiotic compared to when they were used alone. After treatment with CB or Cip with AlyP1400, the observed biofilms that remained attached to the surface of the flow-cell chamber contained a few small micro-colonies. The treatment of AlyP1400 with CB or Cip significantly reduced the biofilm thickness to 16.8 ± 2.20 µm and 13.2 ± 3.42 µm, and surface area to 0.29 ± 0.20 × 10^5^ µm^2^ and 0.24 ± 0.10 × 10^5^ µm^2^, respectively (Table 3). The biovolume was reduced significantly to 5.27 ± 1.34 and 3.37 ± 0.64, respectively (Table 3). The treated biofilms were unstable and less structured (Figure S3e,f) compared to the control biofilms (Figure S3a). These remaining cell aggregates appeared to be predominantly dead, as determined by the overlay of the red fluorescent merged with the green fluorescent (Figure S3e,f). The enhanced efficacy of bacterial killing was especially pronounced for CB and Cip as the membrane compromised cell staining was evident in the micro-colonies of the biofilm. These results were confirmed by the significant reductions in the biofilm thickness, biovolume, and surface area.

## 3. Discussion

*Pseudomonas aeruginosa* biofilm infection has been a substantial public health problem, mainly due to their antibiotic resistance and defiance of the host defense system. This opportunistic pathogen secrets alginate (polysaccharide-like structure), a unique mucoid architecture, which is the main issue in chronic infection treatments, especially with persistent lung infection of CF patients. Attachment to surfaces is required and stress conditions may induce biofilm formation. Although high doses of antibiotics still represent the most successful medical intervention, it unfortunately doesn’t always entirely eradicate the whole bacterial population. Additionally, high levels of antibiotic resistant pathogens usually ensue, hence the development of non-conventional treatment strategies is urgently needed [16,36]. Many attempts to eradicate biofilms have been efficiently used, including biofilm prevention, disruption, or eradication [8,16,19,37,38]. This work demonstrated that the combined effect of AlyP1400 with conventional antibiotics significantly reduced the notorious *P. aeruginosa* biofilm.

Recently, alginate lyase activity received much attention, because the alginate enzymatic degradation expands the potential application of this polysaccharide, not only through the industrial applications, but also for curing life threatening infections [39,40,41]. Extracting alginate lyase enzymes from marine bacteria that possessed alginate depolymerization activity could shift chronic infection treatment strategies. Data shown here confirmed that multiple marine bacterial isolates were able to reduce the alginate solution viscosity by 90%. This is due to the alginate lyase activity, which degrades the alginate to un-saturated uronic acid-containing oligosaccharides via β-elimination of 1,4-glycosidic [42,43]. Targeting mucoid biofilms with the alginate lyase enzymatic activity can reduce viscosity in clinical strains, and in CF specimens, thereby increasing the phagocytosis activities and enhance the antipseudomonal antibiotics’ effects. Consistently, all the supernatants of selected bacterial isolates effectively reduced established *P. aeruginosa* biofilms. These findings matched with previous reports, which demonstrated that several compounds produced by marine bacteria, particularly *Pseudoalteromonas* spp., exhibited potential antibiofilm activities against both Gram-positive and Gram-negative bacteria [40,44,45,46].

Using the purified alginate lyase of *Pseudoalteromonas* sp. 1400 under static and dynamic conditions against *P. aeruginosa* biofilms on polystyrene and glass surfaces, it was revealed that AlyP1400 impaired biofilm development, compared to the non-treated biofilms and led to subsequent biofilm detachment. This may be due to the hydrolysis of *P. aeruginosa* polysaccharides and disruption of the biofilm architecture that increased dispersal of planktonic cells. In previous studies, the lyase enzyme was also shown to reduce the surface hydrophobicity of newly-formed daughter cells shed from the biofilms [39,47].

The most important finding here is that the co-administration of AlyP1400 with Cip or CB increased the killing efficiency of biofilm cells, which was estimated by measuring the biofilm reduction amount and imaging the remaining attached biofilms to the surface of the flow-cell chamber. In contrast, when Cip or CB was used to treat the established biofilm by themselves, a significant reduction was only observed for the biofilm thickness, as the biovolume and surface area of the antibiotics-treated biofilms were not significantly reduced. It has been shown that biofilm-associated *P. aeruginosa* cells are more resistant to antibiotic and these cells have increased response and expression of antibiotic resistance genes [48,49]. It was also shown that the addition of exogenous, purified alginate to non-mucoid (alginate-negative) strains enhanced the resistance to killing by macrophages by providing a protective mucus structure for the bacterial cells [50] Meanwhile, when alginate lyase was combined with liposomal aminoglycoside antibiotics to treat *P. aeruginosa* biofilms, a marked improvement in bactericidal activity was noticed. This may be attributed to shutting down the interaction between the liposomal lipids and the alginate, thus preventing their interaction with the bacterial membrane [32]. Although *P. aeruginosa* produces its own alginate lyase, most of the bacterial lyase enzymes remain in the periplasmic space, and they are only active to assist in expelling the alginate molecules outside the bacterial cells or to assist in releasing cells from the biofilm to colonize a new space [51,52]. There is insufficient evidence regarding the efficiency of *P. aeruginosa* lyase enzyme against its own alginate biofilm. The results of this study provide a promising strategy for biofilm treatment; it can decrease immune evasion and antibiotic resistance of mucoid *P. aeruginosa*, which support the hypothesis that alginate lyases are promising therapeutic candidates.

## 4. Materials and Methods

Sodium alginate (*Macrocystis pyrifera* origin, catalog #A2033) was purchased from Sigma-Aldrich (St. Louis, MO, USA). Bacto Tryptone and Yeast Extract were purchased from Becton and Dickinson (Sparks, MD, USA). The other chemicals were purchased from Fluka Analytical (a Sigma Aldrich company, Oakville, Ontario, Canada).

### 4.1. Bacterial Strains and Growth Conditions

*Pseudomonas aeruginosa* strain PA14, a clinical isolate obtained from a burn patient was used in the biofilm cultivation. Medium and growth conditions for PA14 were performed according to O’May and colleagues [53].

### 4.2. Isolation of Alginate Lyase Producer Strains

The collected marine water samples from Queensland Beach, Nova Scotia. (GPS coordinates: 44°38′05.3″N 64°01′40.6″W) at 22.5 °C and pH 8.11, were used for culturing bacterial isolates with alginate lyase activity, as follows: 10 ml water was shaken in 90 ml enrichment medium (0.5% peptone, 0.1% yeast extract, 0.5% sodium alginate, 3% NaCl, pH 6.5) [53] for 48 h at 28 °C and 150 rpm. Serially diluted cultures were plated on selection medium agar (1% sodium alginate, 0.5% (NH_4_)_2_SO_4_, 0.2% K_2_HPO_4_·3H_2_O, 0.001% FeSO_4_·7H_2_O, 0.1% MgSO_4_·7H_2_O, 3% NaCl, and 1.5% agar, pH 7.0) [54]. Detectable colonies that could grow on selection medium agar were further streak-purified on selection medium agar with 1.8% sodium alginate.

Purified microorganisms were transferred to plates with selection medium and incubated for 48 h at 28 °C. Afterward, the entire plate surface was flooded with three ml of Lugol’s solution and the clear zones around bacterial growth that indicates alginate hydrolysis were measured [54]. Morphologically distinct bacterial colonies with large hydrolytic zones were stored at −80°C in a solution of 40% glycerol with bacterial growth in selection medium broth (1:1 v/v) for further alginolytic activity studies.

### 4.3. Bacterial Identification

DNA from isolated microorganisms was prepared for PCR by boiling an isolated colony in 50% solution of Chelex 100 resin (v/v in sterile water) (Sigma, St. Louis, MO, USA) for 10 min, followed by centrifugation for 10 min at 10,956× *g*. The supernatant was used as a template for PCR method modified from a previously published protocol [55]. GoTaq^®^ Colourless Master Mix (Promega, Madison, WI, USA) was used for the PCRs following the manufacturer’s instructions and contained 0.2 µM of each primer. Universal 16S primers F1 (5’-AGA GTT TGA TCI TGG CTC AG-3’) and R5 (5’-ACG GIT ACC TTG TTA CGA CTT-3’) were used for bacterial isolates [55,56]. PCR products were generated using an initial denaturation step for two minutes at 95 °C followed by 35 cycles of a 60 s 95 °C denaturation, a 30 s 58 °C annealing and a 60 s 72 °C extension. A final extension of 10 min at 72 °C was included. PCR products were visualized on a 1% agarose gel stained with SYBR^®^ Safe DNA Stain (Invitrogen, Carlsbad, CA, USA) under UV light. Upon verifying the presence of the correctly sized single band (1500 bp), the PCR product was cleaned with ExoSAP-IT^®^ (Affymetrix, Santa Clara, CA, USA), following manufacturer’s instructions. The cleaned PCR products were sent to Genewiz (South Plainfield, NJ, USA) for sequencing using the same primers that were used for PCR amplification. For a phylogenetic depiction, the sequenced 16S rRNA genes for each isolate were compared with the GenBank database using the BLAST program through the NCBI’s website [57].

### 4.4. Alginolytic Activity and Substrate Specificity of Cell-Free Supernatants

Bacterial colonies with the highest lyase activity in the clear zone assay above (4.2) were selected for testing their alginolytic activity as follows: three milliliter overnight (16–18 h) bacterial culture (OD at 600 nm adjusted to 1.0) was inoculated into a 97 mL fermentation medium (selection medium with 1.8% sodium alginate) and incubated at 28 °C and 150 rpm for 24 h. After the fermentation and centrifugation for 30 min at 12,096× *g* at 4 °C, cell-free supernatants were filtered through 0.22 µm non-pyrogenic filters and kept at −20 °C.

To measure the viscosity of filtered cell-free supernatants, a Brookfield digital viscometer (model LTVD) was use as per the manufacturer’s protocol. All triplicate samples were measured at room temperature (25 °C) using Spindle No.1 with varied speed rotation (60, 30, and 12 rpm) according to the methods previously mentioned by Pendyala and his colleagues [58] and the non-inoculated fermentation medium was used as a control.

Alginate lyase activities were measured using an absorbance assay at the wavelength of 235 nm. The absorbance of sodium alginate solution was increased due to the formation of a carbon-carbon double bond at the end of the product generated from lyase-mediated cleavage of alginate. Filtered supernatant (20 µL) was added to 180 µL aliquots of 0.1% sodium alginate in 20 mM Tris-HCl, pH 7.0, and was incubated for 30 min at 37 °C. The reaction was terminated by heating all tubes for 10 min at 100 °C. Aliquots of preheated supernatants were run in parallel as a control, and the absorbance of the reaction solution was measured at 235 nm with a spectrophotometer (Eppendorf Bio-photometer Plus). One-unit alginate lyase activity was considered as the amount of enzyme that increased the absorbance by 0.01 per minute. [59]. To test the enzyme specificity, both alginate lyase types, poly-M-specific and poly-G-specific, were distinguishable by white halos and white rings respectively according to the methods reported by Nakagawa and his colleagues [60].

### 4.5. Pseudomonas aeruginosa PA14 Biofilm Cultivation in 96-Microtiter Plate

*Pseudomonas aeruginosa* PA14 cells were cultured in LB broth overnight at 37 °C and 200 rpm. Cells were harvested by centrifugation at 9,798× *g* for 10 min at 4 °C. Pelleted cells were washed three times in phosphate buffered saline (PBS, pH 7.5) and resuspended in M63 medium (1.36% KH_2_PO_4_, 0.2% (NH_4_)_2_SO_4_, 0.02% MgSO_4_·7H_2_O, and 0.2% glycerol with pH 7.0) supplemented with 0.5 mg/L FeSO_4_·7H_2_O, 0.5 µg/mL vitamin B1, and 1.0 µg/mL l-arginine [61]. Each well of 96-microtiter plate (polyvinylchloride plastic) was seeded with 190 µL M63 medium and 10 µL PA14 overnight culture diluted in M63 medium (OD_600_ = 0.5). After incubation statically at 37 °C with 95% relative humidity for 24 h, the medium was removed, the biofilm was fixed with 200 µL 99% methanol for 15 min and then stained with 200 µL of 1.0% filtered crystal violet for five minutes on orbital shaker at room temperature (27 ± 2 °C). Excess stain was removed, and the wells were washed three times with distilled water and dried before biofilm solubilization. Biofilms in each well were dissolved by 200 µL absolute ethanol for 30 s, then 125 µL of dissolved biofilm was transferred into a new 96-well microtiter plates and analyzed using a plate reader (Bio-Rad Benchmark Plus UV-Visible Plate Reader microplate spectrophotometer) at 570 nm [62,63]. Wells with sterilized medium were used as blank controls.

### 4.6. Pseudomonas aeruginosa PA14 Biofilm Inhibition by Cell-Free Supernatants of Pseudoalteromonas and Cellulophaga spp.

Using the biofilm assay in 96-microtiter plate as described above, each well was seeded with 95 µL M63 medium and 5 µL of PA14 overnight culture diluted in M63 medium (OD_600_ = 0.5). The plate was incubated at 37 °C with 95% relative humidity for 48 h. Afterward, each well was topped up with 100 µL of filter-sterilized cell-free supernatant of seven selected *Pseudoalteromonas* and *Cellulophaga* spp. and was incubated for another 24 h or 48 h (two plates in parallel, one for each treatment length). After incubation, the biofilms were then fixed, stained, washed, solubilized and analyzed on at plate reader at 570 nm as described in the previous section.

### 4.7. AlyP1400 Production and Purification

In order to extract sufficient quantities of lyase, we cultured isolate 1400 under different temperatures, covering the range of 10–45 °C, and pHs, covering the range of 4.0–10 in order to determine the optimum culture conditions to purify the enzyme. Both the total and specific alginate lyase enzyme activities were measured as described by Zhu and colleagues [59], and the temperature and pH resulting in the highest enzyme activities were utilized for the following purification procedures. After 24 h fermentation of selection medium with 1.8% sodium alginate and pH 8 with *Pseudoalteromonas* sp. 1400 at 25 °C and 150 rpm, the cell-free supernatant was collected and fractionated with ammonium sulfate between 60% and 80% saturation. The protein was precipitated, resuspended in 3.0 mL of Tris-HCl (20 mM, pH 7.5) and dialyzed against 100 volumes of Tris-HCl (20 mM, pH 7.5), at 4 °C (changed eight times over a 16 h period) using 12–14 KDa molecular-weight-cut-off dialysis tubing (Baxter, Spectra/POR molecular porous membrane tubing). The salt-free protein sample was freeze-dried using a freeze-dryer (Sharp Freeze-110, AAPPTec) and the powder was dissolved in Tris-HCl buffer (20 mM, pH 7.5) (4% w/v as a final concentration) to generate an enzyme solution for further analysis and testing. From this solution, both the total and specific alginate lyase enzyme activities were again measured as described by Zhu and colleagues [59], meanwhile protein contents were evaluated according to BCA protein assay kit (Thermo Scientific Pierce BCA Protein Assay Kit) following the manufacturer’s instruction. The enzyme solution was applied to a DEAE Sepharose column (Sigma-Aldrich,16.0 × 2.0 cm) after equilibration with the Tris-HCl buffer (20 mM, pH 7.5), then was eluted by the same buffer with linear gradient of 0.1 to 0.5 M NaCl at a flow rate of 500 µL/min [64]. Each fraction was collected and measured for enzyme activity and for protein content as described above. Fractions that showed the highest alginolytic activity were pooled, and their purity and molecular mass were detected by sodium dodecyl sulfate polyacrylamide gel electrophoresis (SDS-PAGE) and Zymography [65,66]. The pooled fractions with high alginolytic activity from the DEAE Sepharose column were freeze-dried and dissolved in Tris-HCl buffer (4.0% w/v as a final concentration) and applied into a Sephadex G100 column (Sigma-Aldrich, 100 × 1.6 cm) that was equilibrated with the same buffer and eluted at a 500 µL/min flow rate. After determining alginate lyase activity, detecting protein content and assessing purity by SDS-PAGE, the purified alginate lyase was stored at −20 °C.

### 4.8. AlyP1400 Characterization

Alginate lyase thermo-stability was determined by incubating the purified enzyme 60 µg protein/mL with sodium alginate (0.1%) in Tris-HCl buffer (pH 7.0) at different temperature (10–45 °C). The effect of pH on the enzymatic activity was determined after incubating the purified enzyme at 30 °C (the optimal temperature determined as above) with sodium alginate (0.1%) in buffers with different pH, using phosphate-citrate (pH 3.0–5.0), Tris-HCl (pH 6.0–9.0) or Na_2_HPO_4_-NaOH (pH 10.0) [67]. Both the enzyme thermal stability and pH stability were detected as the relative enzyme activity by using the total enzyme activity assay, as mentioned above, where the highest measured enzyme activity was considered 100% and all other measurements were compared relative to that highest amount.

### 4.9. Liquid Chromatography Tandem-Mass Spectrometry (LC-MS/MS) Enzyme Analysis

The purified alginate lyase was resolved on a 10% SDS separating gel using the SDS-PAGE analysis. The excised gel slices stained with Coomassie blue G-250 were processed for LC-MS/MS as previously described [68], with minor modifications. Briefly, gel slices were rinsed for 2 h in dH_2_O and then cut into ~1.0 mm cube and rinsed twice with 200 µL of dH_2_O. Gel cubes were reduced with 10 mM dithiothreitol (DTT) at 56 °C for 30 min, alkylated with 55 mM iodoacetamide for 30 min at room temperature in the dark, and dehydrated with 200 µL acetonitrile (ACN). Dried gel cubes were saturated with 20 µg/mL of trypsin protease (Pierce Thermo Scientific) for 2 h, then 20 µL of 50 mM ammonium bicarbonate was added and the samples were incubated overnight at 37 °C. Digested peptides were extracted from the gel cubes by treatment with 100 µL of 50% ACN with 5% formic acid. The peptide-containing solution was dried to a pellet in a vacuum centrifuge and subsequently resuspended in 20 µL of a 3% ACN, 0.5% formic acid solution.

The samples were transferred to a 300 µL HPLC vial and subject to analysis by LC-MS/MS on a VelosPRO orbitrap mass spectrometer (Thermo Fisher Scientific) equipped with an UltiMate 3000 Nano-LC system (Thermo Fisher Scientific). Chromatographic separation of the digests was performed on PicoFRIT C18 self-packed 75 mm x 60 cm capillary column (New Objective, Woburn, MA) at a flow rate of 300 µL/min. MS and MS/MS data were acquired using a data-dependent acquisition method in which a full scan was obtained at a resolution of 30,000, followed by ten consecutive MS/MS spectra in both higher-energy collisional dissociation (HCD) and collision-induced dissociation (CID) mode (normalized collision energy 36%). Internal calibration was performed using the ion signal of polysiloxane at m/z 445.120025 as a lock mass. Raw MS data were analyzed using Proteome Discoverer 2.1 (Thermo Fisher Scientific). Peak lists were searched in Uniprot’s Pseudoalteromonadaceae database as well as the cRAP database of common contaminants (Global Proteome Machine Organization). Cysteine carbamidomethylation was set as a fixed modification, while methionine (Met) oxidation, N-terminal Met loss, and phosphorylation on serine, threonine, and tyrosine were included as variable modifications. A mass accuracy tolerance of 5 ppm was used for precursor ions, while 0.02 Da for HCD fragmentation or 0.6 Da for CID fragmentation was used for product ions. Percolator [69] was used to determine confident peptide identifications using a 0.1% false discovery rate (FDR).

### 4.10. Inhibitory Effect of AlyP1400 against P. aeruginosa PA14 Biofilm

Alginate production by PA14 was evaluated after 48 h incubation at 37 °C in 96-microtiter plate filled with M63 medium. Alginolytic hydrolytic activity of AlyP1400 was assayed by incubating 60 µg/mL AlyP1400 with PA14 under the same conditions. A monoclonal antibody MAb F429 (kindly provided as a gift by Dr. Gerald Pier, Harvard Medical School, Boston, Massachusetts) was used to stain the PA14 biofilms recovered from the microtiter plate (with or without AlyP1400) as described previously [70]. Briefly, smears of 100 µL of recovered biofilms were air-dried and fixed with methanol 99% on glass slides, washed and stained for alginate production by immunofluorescence using MAb F429 at 1 mg/mL or a control solution lacking the primary antibody. A non-alginate producer (*E. coli* TOP10, Invitrogen; Cat# LSC404010) was used as negative control. After incubation 1 h at 37 °C, the smears were washed three times with phosphate-buffered saline and covered with 100 µL of 1:500 dilution of donkey anti-rabbit immunoglobulin G Alexa Fluor 488 (Molecular Probes/Invitrogen, Carlsbad, CA) and incubated at 37 °C for 1 h in the dark. After washing, the smears were visualized for alginate production using fluorescence microscope (Model Zeiss LSM 510, Carl Zeiss, Germany) with argon (488 nm) used with an oil-immersion 63x objective.

After confirming PA14 alginate production and AlyP1400 alginate lyase activity on the alginate, the inhibitory effects of AlyP1400 with and without antibiotics was assessed against established biofilms formed in dynamic conditions. To establish the biofilms, overnight cultures of PA14 in LB were centrifuged and washed three times in PBS. Cells were resuspended in M63 medium and adjusted to OD_600_ = 0.5. Three channel flow-cell (1 × 40 × 44 mm; BioCentrum, DTU, Denmark) was assembled and sterilized flowing the manufacturer’s instructions. Each channel was injected with 250 µL of PA14 cell suspension as prepared above. The whole flow cell system was incubated statically for 2 h at 37 °C. The biofilms were developed on the glass substratum of the flow-cell with 48 h of incubation at 37 °C under dynamic conditions (0.2 mm/s linear flow rate) [71]. To determine the effect of AlyP1400 activity combined with antibiotics against the attached biofilm, the flow-cell was incubated at a static condition for 30 min before each channel was injected with 250 µL of one the following: M63 medium (control), AlyP1400 (60 µg protein/mL in Tris-HCl buffer), CB (100 µg/mL), Cip (12 µg/mL), AlyP1400 with CB (1:1 v/v), and AlyP1400 with Cip (1:1 v/v). Each flow cell was kept under static conditions for another 2 h and then each channel was stained for 30 min with 250 µL, 10 µM Syto 61 Red (Thermo Fisher Scientific, Invitrogen) that stains all living cells and fluoresces red (excitation at 628 nm, emission at 645 nm), followed by 250 µL, 10 µM Sytox Green (Thermo Fisher Scientific, Invitrogen) for 30 min, which stains membrane compromised cells and fluoresces green (excitation at 504 nm, emission at 523 nm) to determine the cell death. Confocal laser scanning microscope (Model Zeiss LSM 510, Carl Zeiss, Germany) with argon (488 nm) and a helium neon laser (543 nm) was used to image the biofilms with an oil-immersion 63x objective. The 488 nm laser and a 500 nm–550 nm band pass emission filter and the 543 nm laser with a long pass 560 nm emission filer were used to excite and detect the stained biofilms. Images (*n* = 10) were scanned sequentially at 488 nm and 543 nm for biofilms after the treatments and were processed using Zen 2009 image software. PA14 clusters were identified as any bacterial assemblages with a thickness more than 10.0 µm. Quantitative analysis of fluorescence microscopic images (captured through Z-stack) obtained from flow cell-grown biofilms was performed with COMSTAT image analysis software [72].

### 4.11. Statistical Analyses

Statistical analyses were performed using GraphPad Prism software (version 7.0; GraphPad Software, Inc, La Jolla, CA, USA). ANOVA one-way and two-way tests were used to determine any statistically significant difference between different experimental conditions and biofilm structural parameters obtained from COMSTAT analysis of CLSM images. Differences were considered statistically significant when *P* < 0.05.

## 5. Conclusions

A polysaccharide lyase AlyP1400, with high activity against alginate substrate, has been purified from marine bacterium *Pseudoalteromonas* sp. 1400. This enzyme had a molecular mass at 23 KDa and revealed a broad pH range for high enzyme activity and thermo-stability with a bifunctional lyase activity for both poly-M and poly-G degradation. AlyP1400 efficiently diminished *P. aeruginosa* PA14 biofilms when combined with conventional antibiotic, as the biofilm thickness, biovolume and surface area were reduced after treatment with carbenicillin or ciprofloxacin combined with the lyase enzyme. This result provides the knowledge basis to develop a combination treatment strategy, which targets alginates or polysaccharide matrices within bacterial biofilms in order to enhance the efficacy of known, clinically used antibiotics.

## Figures and Tables

**Table 1:** Identities and alginate lyase activity of isolated bacterial strains.

No.	The Most Closely Related Bacterial Species Name and Sequence ID from GenBank	Alginate Lyase Activity (Clearing Zone, mm) *
101	*Pseudoalteromonas carrageenovora* IAM 12662 strain ATCC43555T, LT965929.1	12.0 ± 1.4
1002	*Celeribacter halophilus* strain Xmb039, KT986167.1	10.5 ± 2.1
2001	*Shewanella algae strain SFH3,* MG738264.1	9.5 ± 0.7
2005	*Vibrio parahaemolyticus* strain HH101313, MG386398.1	14.5 ± 0.7
2006	*Shewanella algae* strain KC-Na-R1, CP033575.1	6.5 ± 0.7
4001	*Vibrio natriegens* strain AUCASVE5, JQ277719.1	7.0 ± 1.4
4002	*Vibrio alginolyticus* strain QY170324, MF101235.1	6.0 ± 1.4
4003	*Vibrio alginolyticus* strain QY170324, MF101235.1	8.0 ± 1.4
4005	*Vibrio parahaemolyticus* strain SC2, MK308579.1	10.0 ± 1.4
6002	*Vibrio azureus* strain Xmb005, KT986135.1	10.5 ± 2.1
6006	*Vibrio alginolyticus* strain FDAARGOS_114, CP014045.1	3.0 ± 1.4
8009	*Vibrio alginolyticus* strain QY170324, MF101235.1	11.0 ± 1.4
9001	*Vibrio parahaemolyticus* strain CHB-33, KR347290.1	5.5 ± 2.1
9002	*Vibrio alginolyticus* strain CX-72, MH368392.1	4.5 ± 0.7
9003	*Vibrio alginolyticus* strain Val-3, MH879822.1	6.5 ± 2.1
9004	*Vibrio diabolicus* strain FDAARGOS_96, CP014094.1	7.5 ± 0.7
**1400**	***Pseudoalteromonas tetraodonis*** **strain GFC, CP011041.1**	**30.0 ± 1.4**
1401	*Pseudoalteromonas carrageenovora* IAM 12662 strain ATCC43555T, LT965929.1	2.5 ± 0.7
**1403**	***Pseudoalteromonas agarivorans*** **strain SDRB-Py1, MG456901.1**	**27.5 ± 0.7**
1404	*Pseudoalteromonas distincta* strain 20KNS10Z3, MH478310.1	4.0 ± 1.4
1405	*Pseudoalteromonas carrageenovora* IAM 12662 strain ATCC43555T, LT965929.1	11.0 ± 1.4
1406	*Pseudoalteromonas agarivorans* strain DSM 14585, NR_025509.1	6.5 ± 0.7
1407	*Pseudoalteromonas tetraodonis* strain GFC, CP011041.1	2.5 ± 0.7
1408	*Pseudoalteromonas tetraodonis* strain GFC, CP011041.1	11.0 ± 1.4
1410	*Pseudoalteromonas tetraodonis* strain GFC, CP011041.1	12.0 ± 1.4
1412	*Pseudoalteromonas atlantica* strain ECSMB14104, CP023464.1	5.0 ± 0.0
**1413**	***Cellulophaga fucicola*** **strain NN015860, NR_025287.1**	**17.5 ± 1.4**
1414	*Pseudoalteromonas carrageenovora* IAM 12662 strain ATCC43555T, LT965929.1	14.5 ± 0.7
**1416**	***Pseudoalteromonas carrageenovora*** **IAM 12662 strain ATCC43555T, LT965929.1**	**20.5 ± 0.7**
1417	*Pseudoalteromonas carrageenovora* IAM 12662 strain ATCC43555T, LT965929.1	9.5 ± 0.7
1418	*Alteromonas stellipolaris* strain PQQ-44, CP015346.1	10.0 ± 1.4
1419	*Pseudoalteromonas tetraodonis* strain GFC, CP011041.1	13.5 ± 2.1
**1421**	***Pseudoalteromonas carrageenovora*** **IAM 12662 strain ATCC43555T, LT965929.1**	**24.0 ± 1.4**
**1422**	***Pseudoalteromonas carrageenovora*** **IAM 12662 strain ATCC43555T, LT965929.1**	**24.0 ± 1.4**
**1423**	***Cellulophaga fucicola*** **strain NN015860, NR_025287.1**	**21.5 ± 2.1**
1427	*Pseudoalteromonas espejiana* strain ATCC 29659, CP011028.1	7.5 ± 0.7

Notes: The bacterial strains with the largest clearing zones that were further used in this study are shown in bold font. * The mean values of three independent experiments are presented as means ± standard deviation, SD (*n* = 3).

**Table 2:** AlyP1400 purification procedures.

Purification Proceedings	Total Protein (mg)	Total Enzyme Activity (U)*	Enzyme Specific Activity (U/mg Protein)	Purification (Fold)	Yield (%)
Crude enzyme	5126.11 ± 5.35	90565 ± 5.0	17.67 ± 0.018	1.00	100
Fractionation I 60% (NH_4_)_2_SO_4_	1551.33 ± 4.16	53375.33 ± 4.51	34.41 ± 0.92	1.95	59
Fractionation II 80% (NH_4_)_2_SO_4_	85.9 ± 1.13	9123 ± 3.0	106.47 ± 1.4	6.03	10
Anion exchange chromatography (DEAE Sepharose)	32.166 ± 1.78	3902.33 ± 6.81	125.94 ± 7.32	7.13	4.0
Gel-filtration chromatography (Sephadex G-100)	1.17 ± 0.06	410 ± 2.0	342.16 ± 3.84	19.36	0.5

Notes: * One-unit (U) enzyme defined as the amount of enzyme required to increase the absorbance at 235 nm by 0.01 per minute. The mean values of three independent experiments are presented as means ± SD (*n* = 3).

**Table 3:** COMSTAT analyses of remained 48-hour old *P. aeruginosa* PA14 biofilms treated for 2 hours in flow-cell chambers.

Treatments	Average Thickness (µm)	Biovolume (µm^3^/µm^2^)	Surface Area (10^5^ µm^2^)
Non-treated	37.2 ± 3.74	10.27 ± 3.77	5.0 ± 3.5
Carbenicillin (CB)	27.9 ± 4.45 *	10.28 ± 1.26	6.3 ± 4.0
Ciprofloxacin (Cip)	27.7 ± 4.14 *	10.70 ± 1.0	6.1 ± 0.1
AlyP1400	25.3 ± 2.41 *	7.71 ± 3.79 *	2.6 ± 1.6 *
AlyP1400 + CB	16.8 ± 2.20 **	5.27 ± 1.34 **	0.29 ± 0.2 **
AlyP1400 + Cip	13.2 ± 3.42 **	3.37 ± 0.64 **	0.24 ± 0.1 **

Notes: The numbers represent the averages of data from three independent experiments, mean values of 30 images are presented as means ± SD. Carbenicillin (100 µg/mL), ciprofloxacin (12 µg/mL) and AlyP1400 (60 µg protein/mL). Significant differences are indicated by the follows: no asterisk, *P* > 0.05, * *P* < 0.05, and ** *P* < 0.01.

## Supplementary Materials

The following are available online at https://www.mdpi.com/1660-3397/17/5/307/s1, Figure S1: Biofilm biomass formed after 48 h in 96-well microplates, treated with different cell-free supernatants for 48 h at 37 °C under static conditions, Figure S2: Peptide-spectra matches obtained from the in-gel tryptic digestion of AlyP1400 analyzed by LC-MS/MS, Figure S3: Selected representative images of *P. aeruginosa* PA14 biofilms grown under dynamic conditions on glass surface at 37°C, for 48 h under a flow of M63 medium exposed to different treatments.
