# Supplementary material for: Marine Bacteria, A Source for Alginolytic Enzyme to Disrupt Pseudomonas aeruginosa Biofilms
Source: Mar Drugs. 2019 May 24;17(5):307. doi: 10.3390/md17050307 (PMC6562671; doi:10.3390/md17050307)
Supplement: Supplementary file 1 [file marinedrugs-17-00307-s001.pdf]

Supplementary Materials

Marine Bacteria, A Source for Alginolytic Enzyme to Disrupt *Pseudomonas aeruginosa* Biofilms

Said M. Daboor [1,3,*], Renee Raudonis [1], Alejandro Cohen [2], John R. Rohde [1] and Zhenyu Cheng [1,*]

[1] Department of Microbiology and Immunology, Dalhousie University, Halifax, Nova Scotia, B3H 4R2; Canada; r.raudonis@dal.ca (R.R.); john.rohde@dal.ca (J.R.R.)
[2] Department of Biochemistry and Molecular Biology, Dalhousie University, Halifax, Nova Scotia, B3H 4R2; Canada; alejandro.cohen@dal.ca
[3] National Institute of Oceanography and Fisheries, Cairo, 11516, Egypt

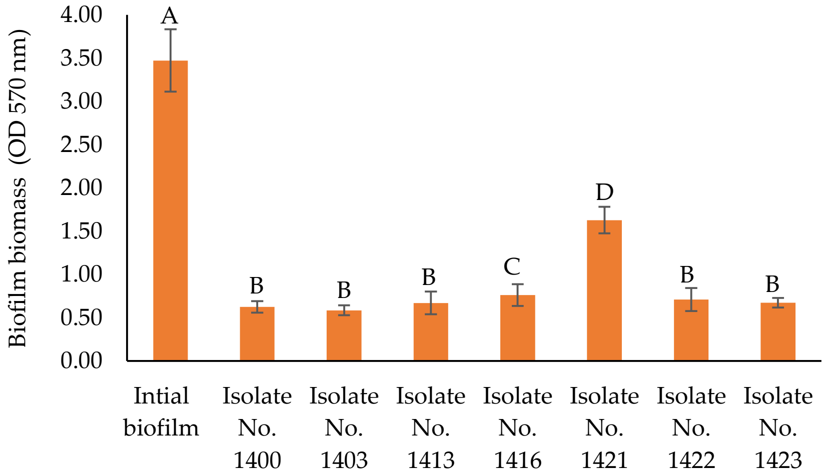

Figure S1: Biofilm biomass formed after 48 h in 96-well microplates, treated with different cell-free supernatants for 48 h at 37 °C under static conditions. Error bars represent standard deviation of three independent experiments. Different letters indicate statistically significant differences between groups ($p < 0.05$).

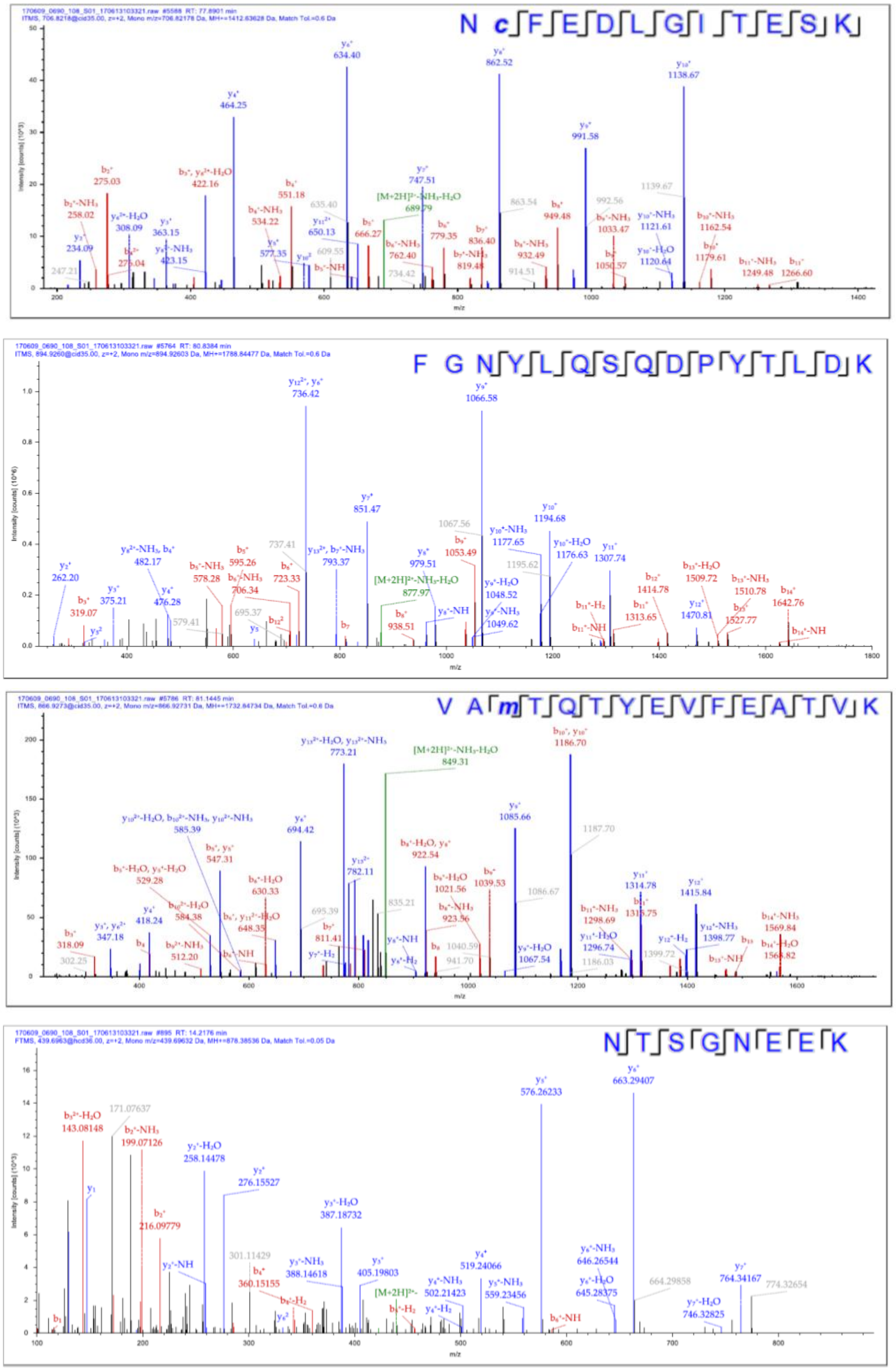

Figure S2: Peptide-spectra matches obtained from the in-gel tryptic digestion of AlyP1400 analyzed by LC-MS/MS. Four representative peptide sequences of alginase (NCBI accession ACB87607.2) are shown, with the diagnostic y- and b- fragment ions (in blue and red, respectively).

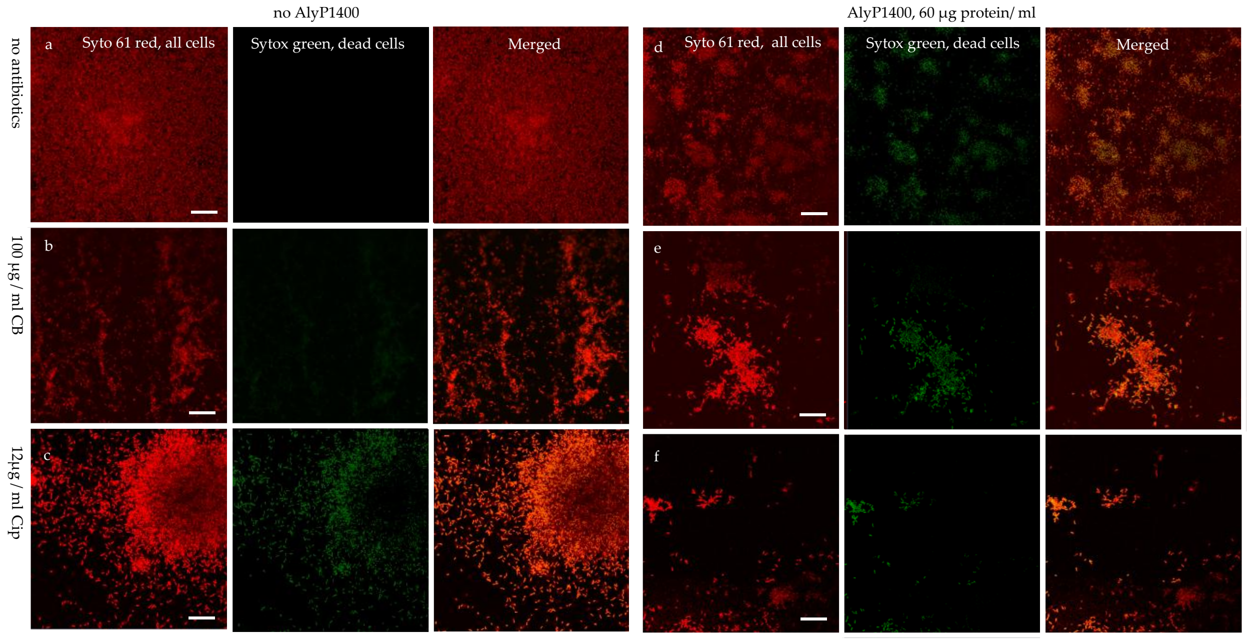

Figure S3. Selected representative images of *P. aeruginosa* PA14 biofilms grown under dynamic conditions on glass surface at 37°C, for 48 h under a flow of M63 medium exposed to different treatments: (a) non-treated, control biofilm; (b) CB (100 µg/mL); (c) Cip (12 µg/mL); (d) AlyP1400 (60 µg protein/mL); (e) combination of AlyP1400 and CB; and (f) combination of Alyp1400 and Cip. Biofilms were visualized with confocal laser scanning microscopy using magnification power 63X. Syto 61 Red fluorescence emission was from all cells, while Sytox Green fluorescence emission was from dead or dying cells, and in the merged image the yellow fluorescence represented a combination of both red and green fluorescence. The images are representative of three independent experiments from which a total of 30 image stacks were obtained. Bars equal 20 µm.
